# Feline Calicivirus Can Tolerate Gross Changes of Its Minor Capsid Protein Expression Levels Induced by Changing Translation Reinitiation Frequency or Use of a Separate VP2-Coding mRNA

**DOI:** 10.1371/journal.pone.0102254

**Published:** 2014-07-09

**Authors:** Maria Haß, Christine Luttermann, Gregor Meyers

**Affiliations:** Institut für Immunologie, Friedrich-Loeffler-Institut, Tübingen, Germany; Penn State University School of Medicine, United States of America

## Abstract

Caliciviruses use reinitiation of translation governed by a ‘termination upstream ribosomal binding site’ (TURBS) for expression of their minor capsid protein VP2. Mutation analysis allowed to identify sequences surrounding the translational start/stop site of the feline calicivirus (FCV) that fine tune reinitiation frequency. A selection of these changes was introduced into the infectious FCV cDNA clone to check the influence of altered VP2 levels on virus replication. In addition, full length constructs were established that displayed a conformation, in which VP2 expression occurred under control of a duplicated subgenomic promoter. Viable viruses recovered from such constructs revealed a rather broad range of VP2 expression levels but comparable growth kinetics showing that caliciviruses can tolerate gross changes of the VP2 expression level.

## Introduction

The members of the family *Caliciviridae* represent nonenveloped viruses that are causative for gastrointestinal diseases in humans and different diseases in animals [1]. Five genera are classified within the family, namely *Lagovirus*, *Sapovirus* and *Nebovirus* or *Vesivirus* and *Norovirus*. Members of the genus *Vesivirus* were the first caliciviruses studied in more detail. Long known members are feline calicivirus, vesicular exanthema of swine virus (VESV), and San Miguel Sea lion virus (SMSV). FCV infection of cats is associated with different respiratory syndromes ranging from mild forms to fatal illness resulting in most cases from systemic infection [reviewed in [2]].

**Figure 1:**
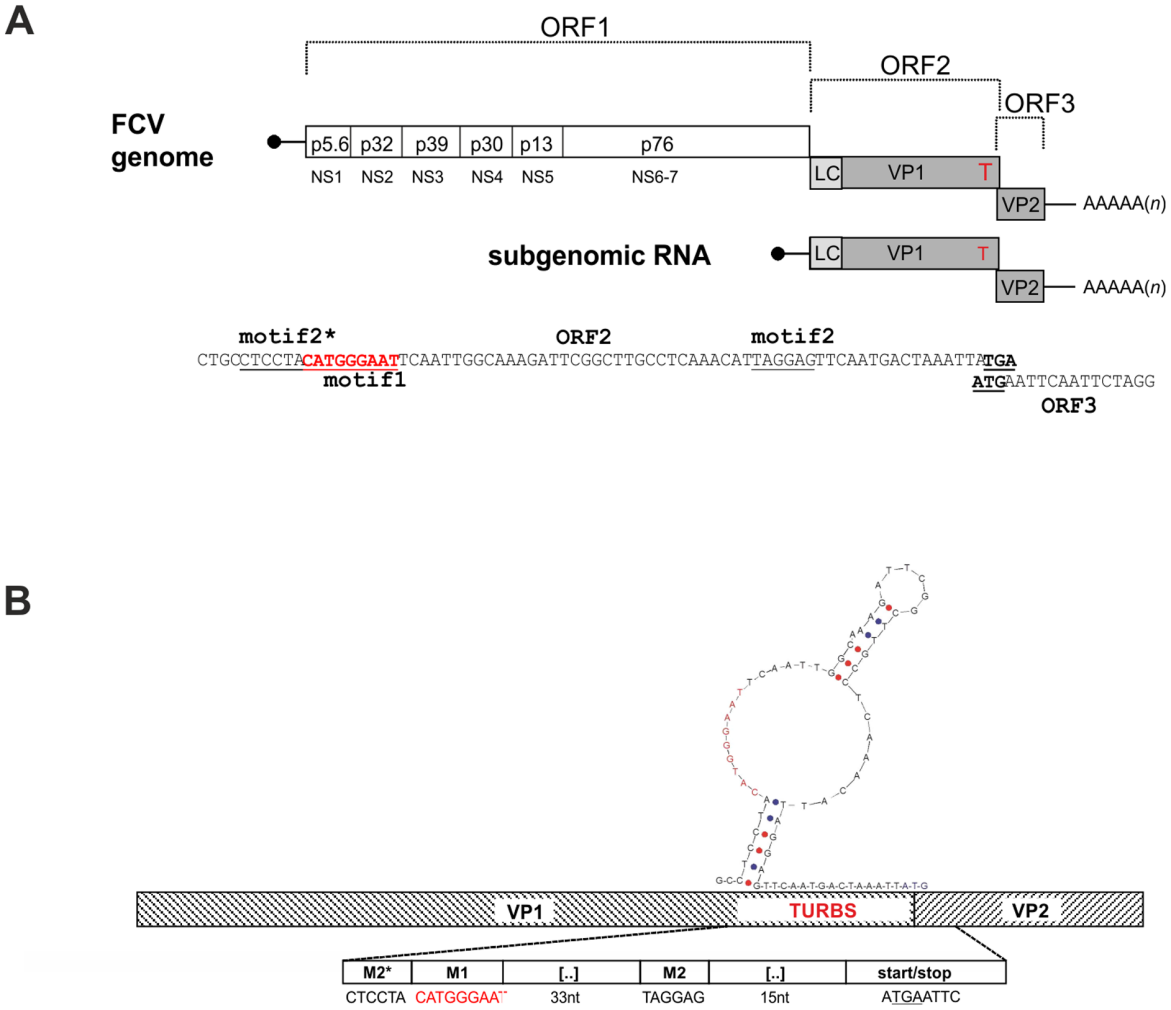
Organization of FCV genome and TURBS. A) Schematic map of FCV genomic and subgenomic RNAs and location of the essential elements of the TURBS in the 3′ terminal sequence of ORF2. On top, the basic organization of the FCV genomic and subgenomic RNAs is illustrated (not drawn to scale). Shaded bars represent ORF2 and ORF3 coding for the capsid proteins and the so-called leader of the capsid protein (LC, light grey bar). The white bar symbolizes the nonstructural protein coding region (ORF1) with the different cleavage products named by size (within bar) and NS protein nomenclature (below bar). The nontranslated regions (NTR) at the 5′ and 3′ end are shown as black lines, the poly(A) tail at the 3′ end is indicated. VPg (virus protein, genome linked) present at the 5′ ends of both the genomic and subgenomic RNAs is symbolized by a black circle at the end of the line representing the 5′ NTR of the RNAs. The proteins encoded by the subgenomic RNA are indicated by abbreviations: LC: Leader of the capsid, VP1: major capsid protein, VP2: minor capsid protein. Red ‘T’ = TURBS. Below the scheme, the 3′ terminal 72 nucleotides of the ORF2 cDNA sequence are shown. Motif 1 (red), 2* and 2, determined by previous analyses [23], and the start/stop codons in the overlap-region of ORF2 and ORF3 are highlighted. B) Blow up of the cDNA region containing the TURBS with the proposed structure of the motif 2*, motif 1 (red) and motif 2 region. G-C and A-T pairing is symbolized by red and blue dots, respectively.

Caliciviruses are positive strand RNA viruses. The genomic RNA is nonsegmented with a length of about 7.5 kb and contains 2 or 3 functional ORFs for members of the genera *Lagovirus*, *Sapovirus* and *Nebovirus* or *Vesivirus* and *Norovirus*, respectively [1]. Only in the murine Norovirus (MNV) a fourth ORF has been found coding for VF1, a protein involved in counteracting the innate immune response of the host cell. VF1 is most likely translated via a leaky scanning like mechanism [3]. The genomic RNA is polyadenylated at the 3′ end and carries a viral protein VPg covalently linked to the RNA 5′ end via a tyrosine residue [4]–[8]. A subgenomic mRNA (sg mRNA) coterminal with the 3′ terminal 2.2 kb of the genome is transcribed in the infected cell. This sg mRNA carries both VPg and a 3′ Poly(A) tail and is packaged into virus particles [8], [9]. Both the major capsid protein VP1 of ca. 60 kDa and the minor capsid protein VP2 of ca. 8 to more than 20 kDa are translated from the sg mRNA [1]. As a unique feature of the genus *Vesivirus* the VP1 protein is translated as a precursor protein (Fig. 1), which is cleaved into the leader protein and the mature capsid protein by a viral protease [10]. 180 copies of VP1 build up the basic structure of the virion [11]. In contrast, the viral particle contains only a few molecules of VP2, so that this protein can hardly be of structural importance for the capsid. Some findings point towards different putative functions of the protein. Indications were found that VP2 negatively regulates the activity of norovirus RNA polymerase [12], regulates expression and stability of norovirus VP1 [13], interacts with VP1 and might assist in genome packaging [14]. In MNV, indications were found that VP2 plays a role in manipulation of the adaptive immune response to the virus [15]. However, there is so far no convincing hypothesis for the fact that this protein is essential for generation of infectious virus particles [16].

The frames coding for the two capsid proteins overlap by 1 to 8 nucleotides [1], [17]. VP2 was shown to be translated from the sg mRNA via a translation termination/reinitiation process that is driven by the so-called TURBS (termination upstream ribosomal binding site), an RNA element of about 40–80 nucleotides located upstream of the start/stop site [2], [18]–[21]. The TURBS region contains three short sequence motifs essential for VP2 translation as determined by deletion mapping (Fig. 1). Motif 1 is conserved among caliciviruses and is located at similar positions in the mRNAs of the different caliciviruses, upstream of the 3′ terminal ORFs. This motif is complementary to the loop region of helix 26 within 18S rRNA [18]. Hybridization of motif 1 to 18S rRNA could tether the ribosome to the viral RNA. Published data indicate that motif 1 also interacts with initiation factor eIF3 [22], a process that could assist the hybridization mediated effect. Motifs 2 and 2* represent stretches of complementary sequences, located directly upstream (motif 2*) or at defined positions downstream of motif 1 (motif 2). The 2*/2 sequences are not conserved with regard to primary sequence so that their ability to form a stem structure seems to be the important point. It was supposed that the secondary structure element established by intramolecular hybridization of these sequences plays a role in positioning of the ribosome relative to the start site of the 3′ terminal ORF [20], [23].

During our analyses, we observed variation of VP2 expression rates in consequence of changes affecting the sequence located downstream of the start/stop site. These findings raised the question about a putative TURBS motif 3 located within the VP2 coding sequence. In the present report experiments are described that focus on the importance of VP2 coding sequences for reinitiation. In addition, the effect of modulated VP2 expression rates on FCV recovery and propagation is analyzed.

## Materials and Methods

### Cells and viruses

BHK-21 cells (kindly provided by T. Rümenapf) were grown in Dulbecco's modified Eagle's medium supplemented with 10% fetal calf serum and nonessential amino acids. Crandell Reese feline kidney (CRFK) cells (ATCC CCL 94) were used for all infection experiments with FCV. These cells grown at 37°C are commonly used for propagation of FCV. The use of other cells and incubation at different temperatures were shown to result in quantitative changes with regard to FCV replication efficiency and individual other features of virus propagation [24], [25] but did not lead to qualitative differences. CRFK cells were grown in Dulbecco's modified Eagle's medium (DMEM) supplemented with nonessential amino acids and 10% fetal calf serum (FCS).

Vaccinia virus MVA-T7 [26] was kindly provided by B. Moss (NIH, Bethesda, MD) and the FCV vaccine strain 2024 by K. Danner, Hoechst Roussel Vet GmbH.

### Construction of recombinant plasmids

Restriction and subcloning were done according to standard procedures. Restriction and modifying enzymes were purchased from New England Biolabs (Schwalbach, Germany) and Fermentas GmbH (Sankt Leon-Rot, Germany).

pCH and pMH-constructs were established on the basis of plasmid pCH1 [23]. Full length -constructs were generated on the basis of pIK12 [27]. pVP2 was generated by subcloning of ORF3 into the pCI vector (Promega).

Point mutations and deletions were introduced by standard PCR-based site-directed mutagenesis methods using thermostable *Pfu* polymerase (Promega, Heidelberg, Germany) and synthetic primers purchased from Invitrogen (Karlsruhe, Germany) or Metabion (Munich, Germany). The cloned PCR products were all verified by nucleotide sequencing with the BigDye Terminator Cycle Sequencing Kit (PE Applied Biosystems, Weiterstadt, Germany). Sequence analysis and alignments were done with Genetics Computer Group software [28]. Details of the cloning procedure and the sequences of the primers are available on request.

### Expression, detection, and quantification of proteins in the mammalian cell system

Transient expression of plasmids in BHK-21 cells using vaccinia virus MVA-T7, metabolic labeling with [^35^S]methionine or [^35^S]cysteine (ICN, Eschwege, Germany), preparation of cell extracts, and recovery of immunoprecipitates with double precipitation were done as described previously [23]. Briefly, VP2 expression efficiency was quantified after SDS-PAGE separation of VP1 and VP2 precipitated with antisera V1 [27] and V2 (raised against bacterially expressed VP2), respectively. Double precipitation was used to ensure quantitative recovery of the proteins as tested before [23]. The precipitates were combined, and aliquots thereof were separated by 10% PAGE. The gels were analyzed with a Fujifilm BAS-1500 phosphorimager, and the intensities of the signals were determined with TINA 2.0 software (Raytest, Straubenhardt, Germany). The molar ratio of VP1 and VP2 was calculated based on the number of labeled residues within the proteins and the measured radioactivity. For comparison of expression efficiencies of different constructs, the VP2 expression level of the wild-type (wt) construct pCH1 was defined as 100%. The amounts of VP2 expression of the other constructs were normalized to the values determined for VP1 as an internal standard. The normalized value for VP2 was then used for calculation of the expression efficiency, given as a percentage of the wt value. The data presented here represent the average of at least three independent experiments.

For protein analysis in cells infected with FCV or mutants thereof CRFK cells were infected with the desired virus for 2 h, followed by 1 h incubation in serum without methionine and label with medium containing [^35^S] methionine for 8 h. Further analysis was done as described above.

### Recovery and analysis of FCV mutants

Recovery of infectious FCV from cloned cDNA and recording of growth curves were done as described before [27]. Briefly, CRFK cells were seeded to 80% confluence in 3.5-cm dishes, infected with MVA-T7 for 1 h (kindly provided by B. Moss, National Institutes of Health, Bethesda, Md.), and transfected with pJT (FCV full length cDNA clone [27]) or mutants thereof. The cells were incubated with the transfection mixture (Lipofectamine 2000, Invitrogen, Karlsruhe, Germany) for 4 h and incubated for another 16 h in DMEM with 10% FCS. After this incubation time, a pronounced cytopathic effect (CPE) was visible that was at least mostly due to MVAT7, since it was also detectable in mock-transfected cells (data not shown). Cells together with supernatants were subjected three times to freeze-thawing, passed through a 0.1-µm-pore-size sterile filter (MILLEX-VV; Millipore Products, Bedford, Mass.) to eliminate the vaccinia virus [29]. One hundred microliters of filtrate from each transfection reaction mixture were used to infect CRFK cells. Recovery of infectious virus was monitored by detection of characteristic FCV CPE. Growth curves were recorded after infection of CRFK cells with a multiplicity of infection of about 0.0003 and were conducted with viruses of the 6^th^ passage, checked for stability of the introduced mutation by sequence analyses. Detection of viral RNA by Northern blot was done as described in [20].

The VP2 trans-complementation assay was conducted analogous to virus recovery (see above). For transfection two plasmids were used, a full length clone and a VP2 expression construct (pVP2). Cotransfection was done with a full length clone containing 3 stop codons in the VP2 coding sequence and a pVP2 construct displaying either the wt sequence (control) or the desired mutation for complementation. Constructs were equivalent to the ones published by Sosnotsev et al. [16]. Alternatively, a full length construct was used that lacked the 3 stop codons but contained the mutations to be analyzed and was complemented with wt VP2. Filtrates were transferred to a fresh CRFK monolayer and checked for infectious virus by detection of CPE.

## Results

### Sequences downstream of the start/stop site influence VP2 expression efficiency

**Figure 2:**
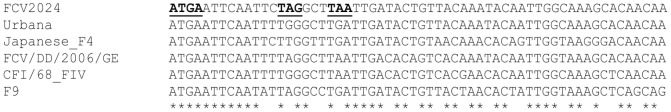
Comparison of the 5′ terminal VP2-coding sequences of different FCV isolates. Comparison of the cDNA sequences of the ORF3 5′ region for different FCV isolates. In the FCV 2024 sequence, the start/stop site and the two downstream stop codons in the ORF2 frame are highlighted. Residues conserved in all sequences are marked by asterisks.

Expression of the minor capsid protein VP2 occurs via a translation termination/reinitiation mechanism governed by the TURBS located upstream of the start/stop site within the region coding for the major capsid protein VP1. Earlier experiments conducted with sequences from the rabbit haemorrhagic disease virus (RHDV) and FCV indicated that mutations downstream of the start/stop site had an influence on the reinitiation frequency. Comparison of the nucleotide sequences of this region displayed by different FCV isolates revealed considerable conservation with a stretch of 11 residues including the start/stop site identical in all sequences (Fig. 2). This finding points at a possibly important role of the primary sequence here. We therefore conducted a systematic search for mutations within the region downstream of the start/stop site in the FCV RNA influencing VP2 expression.

**Figure 3:**
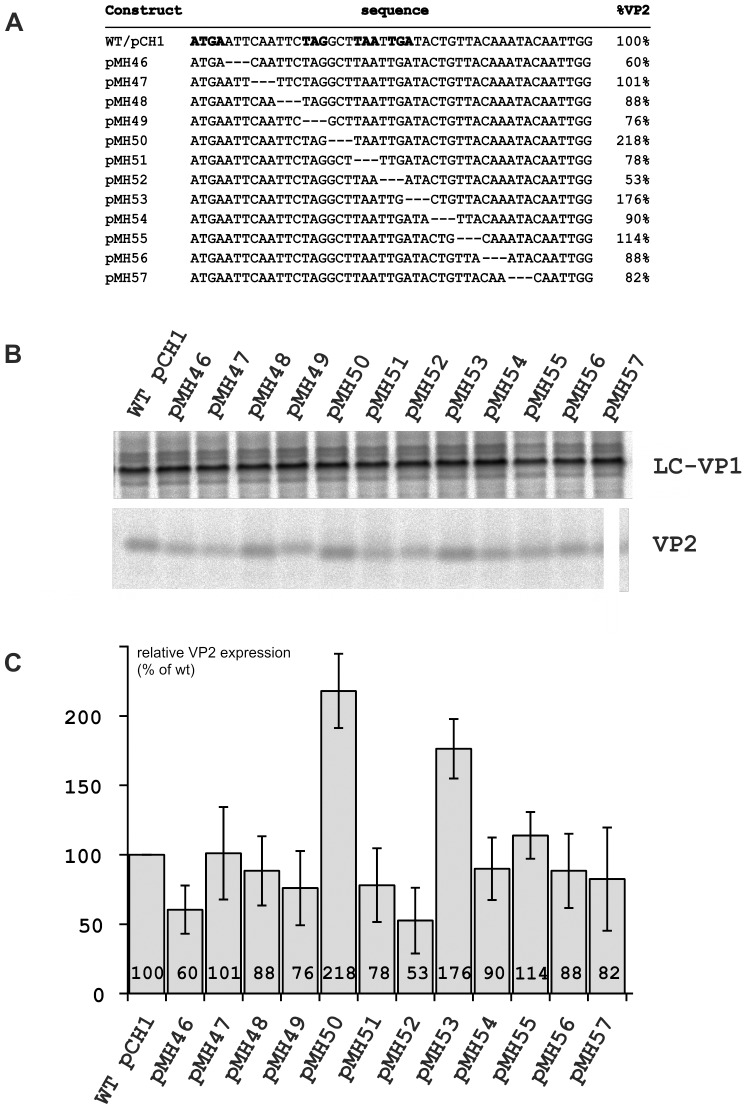
Effect of three nucleotide deletions downstream of the start/stop site on translation reinitiation. A) cDNA sequences of wild type pCH1, a cDNA construct designed for expression of FCV ORFs 2 and 3 [23], and mutants thereof with deletions of 3 nucleotides within the 5′ part of ORF3. On the left, the name of the construct is given and on the right the VP2 expression level compared to the wt construct is given. In the wt sequence, the start/stop site and the downstream stop codons are highlighted. B) Autoradiographs show the proteins immunoprecipitated after transient expression of the constructs indicated on top. On the right site the name of the precipitated proteins is given. Slight variation in the electrophoretic mobility of the different VP2 proteins was observed which is especially obvious for the pMH49 product. According to nucleotide sequence analyses these differences are not due to unwanted second site changes and therefore reflect the differences in amino acid composition between the individual mutated proteins.C) The VP2 expression efficiency is given in a bar diagram with the standard deviation indicated by error bars. The presented data represent the mean values of three independent experiments given as the percentage of the value determined for pCH1 (data normalized relative to the levels of LC-VP1 expression).

As a first step, we established in our standard expression construct (pCH1, [23]) a set of mutants with 3mer deletions in the 36 nucleotides following the VP1 stop codon (Fig. 3). Plasmid pCH1 allows the expression of an RNA equivalent to the FCV sg mRNA so that it allows to study the influence of various mutations on the translation reinitiation leading to VP2 expression. Transient expression analyses revealed nearly wt levels of VP2 expression for most of the constructs containing the deletions (ca. 80 to 115% of wt level) (Fig. 3). Constructs pMH46 and pMH52 showed a moderate reduction of the VP2 level by a factor of about 2. Interestingly, we also found two mutants (pMH50 and pMH53) expressing significantly higher amounts of VP2 than the wt.

**Figure 4:**
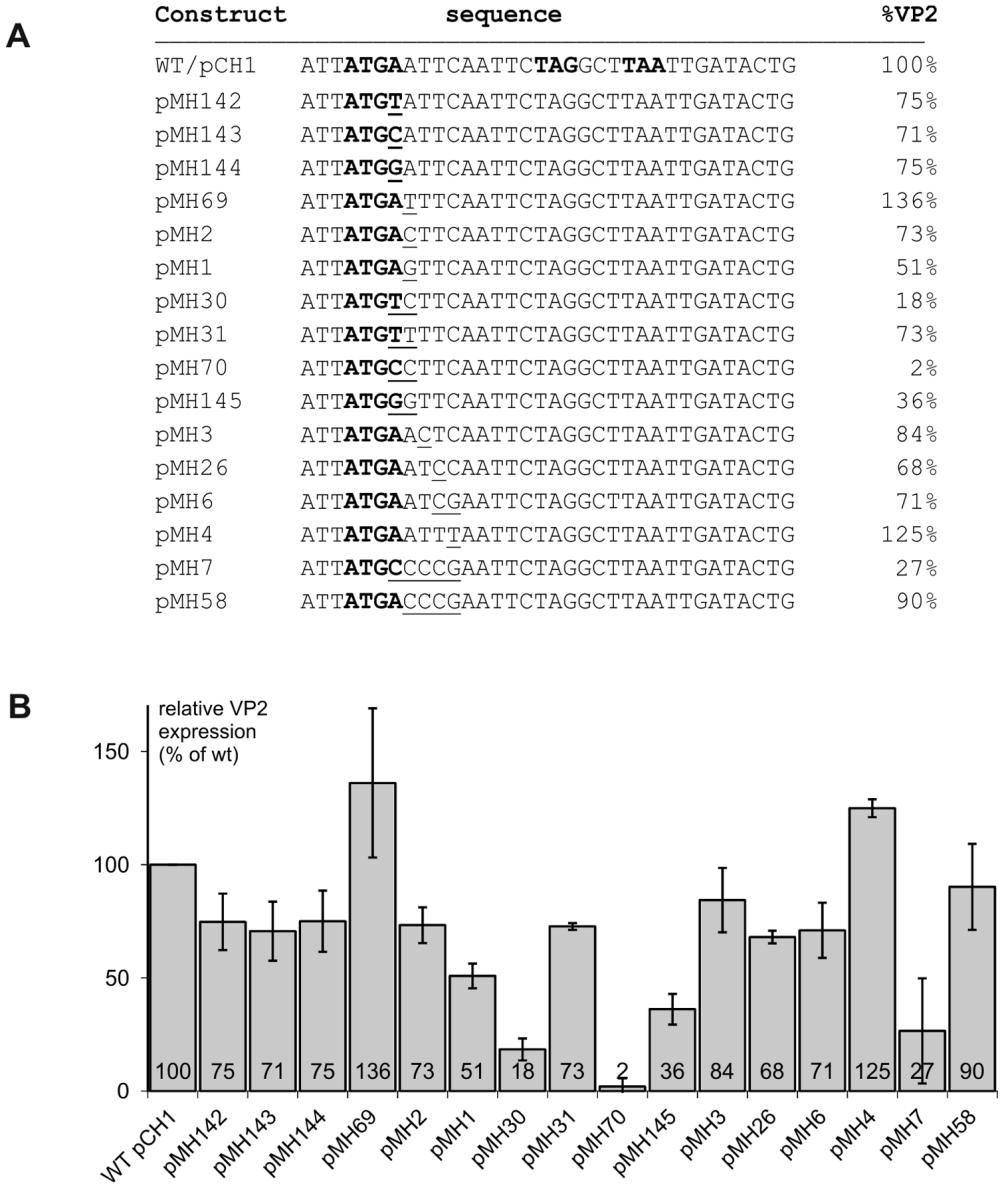
Influence of point mutations shortly downstream of the start/stop site on reinitiation frequency. Results of transient expression studies with mutants of the sg mRNA expression construct pCH1 (wt) containing exchanges within the first 5 residues of ORF3. Panel (A) shows the names (left column) and sequences (middle column) of the different constructs with the start/stop site ATGA in bold face and the mutated residues underlined. For pCH1 the two stop codons in the ORF2 frame downstream of the start/stop site are also given in bold face. The right panel gives the VP2 expression rates as % of the wt (wt set to 100%) normalized to the VP1 expression rate. Panel (B) summarizes the expression rates of the different constructs determined via transient expression, *in situ* labelling with ^35^S amino acids, immunoprecipitation and phophorimager quantification in a bar diagram. The results given represent the mean values of at least three independent expression experiments and indicate the expression levels in % of the wt construct (wt set to 100%), normalized to the expression level of LC-VP1. Error bars give the standard deviation.

Further mutation analysis concentrated on the first 5 nucleotides downstream of the VP2 start codon (Fig. 4). A variety of point mutations was introduced. Several exchanges affecting the first two residues downstream of the AUG had major effects on VP2 expression. All of these mutants changed the stop codon into a coding triplet, so that the effects might in part result from loss of the termination function that is known to be essential for reinitiation [23]. However, we have shown before that the two stop codons following at positions 4 and 6 downstream of the original termination signal can efficiently compensate for the loss of the stop function in the start/stop site [23]. This is also seen in the experiments presented here where all three changes of the A in the UGA codon preserved reinitiation frequencies of 71 to 75% (pMH142-144). In contrast, a combination of one of the latter changes with a mutation of the following residue had major impact in most cases with the strongest reduction to only 2% of the original VP2 expression level resulting from introduction of a CC dinucleotide instead of the AA in the wt sequence (pMH70). Similarly, GG or TC at these positions reduced the VP2 yield considerably more than the corresponding single exchange of the last residue of the UGA codon alone. Since single exchanges of the A residue following the UGA codon had again not the same effects as the double exchanges (see results for pMH69, pMH2 and pMH1) it can be concluded that the combination of the two changes is crucial for the strong effects. However, it has to be stressed that the AA doublet is not *per se* important for high reinitiation frequency since construct pMH31 with an exchange of these two residues for TT leads to expression of VP2 with 73% of wt level. It has also to be mentioned that the sequence context seems to be of major importance for the effect of mutations at this site since the CCCC in pMH7 has a lower effect than the CC in pMH70 (Fig. 4).

### Downstream translational termination codons modulate VP2 expression via a sequence effect

**Figure 5:**
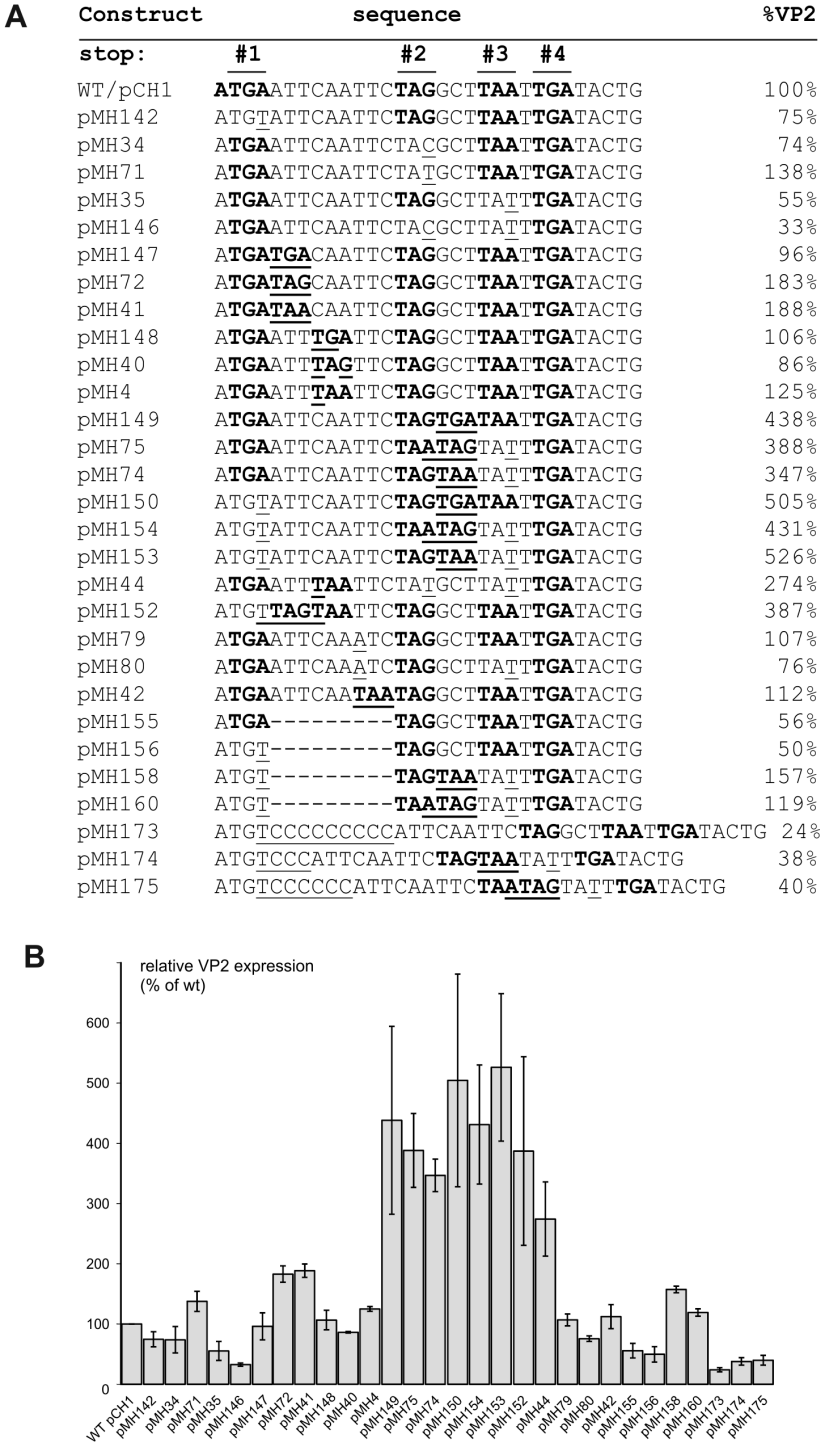
Effect of number and location of stop codons downstream of the start/stop site on VP2 expression levels. Results of transient expression studies with mutants of the sg mRNA expression construct pCH1 containing exchanges affecting the number and/or position of stop codons within the 5′ part of the ORF3 containing RNA. In (A) the sequences of the respective parts of the constructs are shown in the middle column. All stop codons in the given sequences are shown in bold face. Nucleotides differing from the wt sequence are underlined. For the wt pCH1, also the start codon of the start/stop site is given in bold face and the number of the stop codon is given above of the sequence. The names of the constructs and the determined VP2 expression levels are listed in the left and right columns, respectively. In (B) the results of transient expression studies conducted with the constructs are shown. The reinitiation frequency was determined via immunoprecipitation of the transiently expressed proteins, *in situ* labelled with ^35^S amino acids, and phophorimager quantification. The results given represent the mean values of at least three independent expression experiments and indicate the expression levels in % of the wt construct (wt set to 100%). The results were normalized to the amount of LC-VP1 to correct for variations in transfection rates. Error bars indicate standard deviation. Please note that stop codon #4 located closest to the 3′ end in the displayed part of the pCH1 sequence is in another reading frame than the first 3 termination codons.

Earlier studies had shown that mutation of the two in frame stop codons (stop codons #2 and #3) downstream of the termination codon in the start/stop site (stop codon #1) reduced the reinitiation frequency by almost 60% [23]. To get an idea of the role of these codons for VP2 expression, we conducted a systematic mutation analysis (Fig. 5).

As a first step, we eliminated stop codon #1 (pMH142), stop codon #2 (pMH34, pMH71), stop codon #3 (pMH35), or both #2 and #3 (pMH146). Except for the inactivation of stop codon #2 in pMH71 that resulted in a moderate increase of VP2 expression to 138% of wt level, all the mutants showed reduced VP2 levels ranging from 33 to 75%. These results indicated that in general the presence of more than one stop codon in frame with the VP1 coding sequence enhances VP2 yields, even though some bias from the sequence context seems to play a role. The latter point is obvious from comparison of pMH34 and pMH71 that both knock down stop codon #2 but by different single base substitutions at the same position. Whereas pMH71 shows increased VP2 levels, pMH34 results in a lower expression rate.

For further analysis, we introduced a third in frame termination signal at different positions between stop codon #1 and #2 (constructs pMH147, pMH72, pMH41, pMH148, pMH40, and pMH4). All these changes resulted in more or less wt levels of VP2 translation with the exception of pMH41 and pMH72 carrying the TAA and TAG codons just downstream of stop codon #1, respectively, that yielded about double amounts of VP2.

As a next step, we wanted to see, whether the introduction of an extra termination signal between stop codon #2 and #3 has an effect on VP2 expression. We therefore established construct pMH149 with the triplet located between stop codons #2 and # 3 changed to TGA. Surprisingly, the expression analysis revealed a highly enhanced VP2 level of 438% for this construct. This effect seems to be not dependent on the presence of a higher number of termination signals in general, but more due to a position effect, since replacement of the original stop codon #3 in a similar construct still resulted in highly elevated VP2 levels (constructs pMH74 and pMH75 with VP2 levels of 347% and 388%, respectively). This position effect becomes even more obvious when constructs pMH150, 153 and 154 are regarded, in which a mutation of stop codon #1 of the start/stop site is combined with downstream termination signal combinations as before, resulting in very high levels of VP2 expression of up to 526% of the wt. Similarly, construct pMH152 shows higher VP expression than pMH41 (387% versus 188%, respectively) although the stop of the start/stop site together with a newly introduced TAA have been moved downstream by one codon position in pMH152.

Other data supporting the conclusion that not the mere number but the position of the stop codons as well as the general sequence context are responsible for the reinitiation frequency come from analysis of constructs pMH44, for which a VP2 expression level of 274% was determined despite the loss of one stop codon. In fact, the two downstream stop codons #2 and #3 were deleted here and a new termination signal was introduced two codons downstream of the start/stop site. It has to be noticed that this construct is equivalent to pMH4 except for the deletion of stop codons #2 and #3. Compared to pMH4, removal of two termination signals resulted in doubling of the VP2 level here which can only be explained by a sequence context effect.

Similarly, translocation of the stop codons #2 and #3 by deletion of the 9 nucleotides following stop codon #1 as in pMH155 or in pMH156, the corresponding construct with a mutation of stop codon #1, reduced VP2 translation efficiency significantly. An equivalent effect was also seen for pMH158 and 160, two constructs obtained by deletion of the above mentioned 9 nucleotides from pMH153 and 154, respectively. These constructs revealed an about threefold reduction of VP2 levels compared to the parental constructs pMH153 and pMH154 (Fig. 5).

Also the insertion of 3, 6 and 9 nucleotides in pMH174, pMH175 and pMH173 influenced the VP2 expression rate compared to the corresponding parental constructs pMH153, pMH154, pMH142. For the pair pMH174 and pMH153, the change was more than a factor of 10 (38% versus 526%, respectively) (Fig. 5), whereas for pMH173/pMH142 only a threefold difference was identified and for pMH175 the insertion of 9 C residues with respect to pMH154 had no effect at all (Fig. 5). These findings argue again for the importance of the sequence context and the position of the stop codon for the putative termination effect.

**Figure 6:**
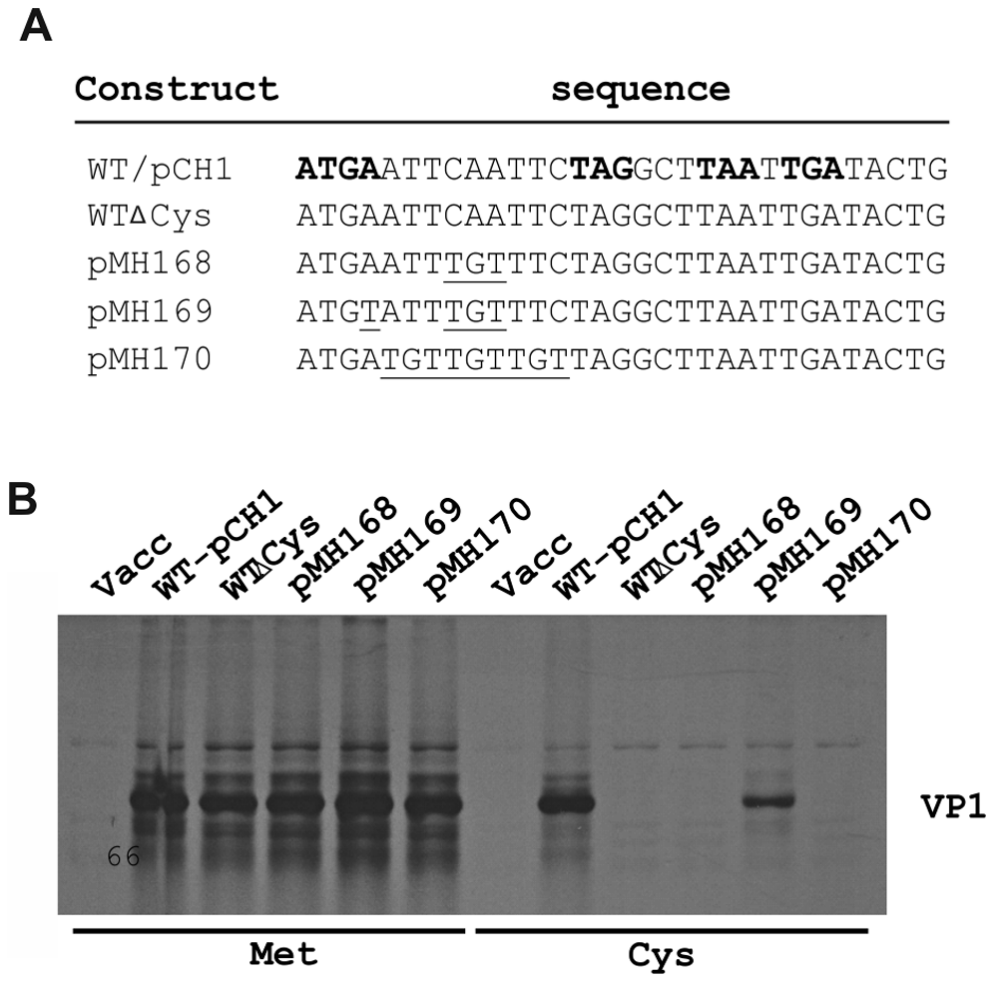
Analysis of putative translational readthrough at the start/stop site. The results of transient expression studies aiming at detection of potential translational readthrough at the termination signal within the start/stop site are shown. The experiments were based on a synthetic expression construct, in which all Cys codons within the VP1-coding sequence were altered into Ser codons (WTΔCys). This construct and mutants thereof were analysed in transient expression studies. The mutants pMH168 and pMH170 were established by introducing Cys codons in the VP1 frame downstream of the termination signal of the start/stop site of WTΔCys as shown in the sequence in (A) (altered nucleotides underlined). Construct pMH169 served as a control. It was generated by changing the stop codon of the start/stop site in pMH168 into a Cys codon. For the wt construct pCH1, the start/stop sequence and the stop codons downstream thereof are given in bold face. In (B) the results of transient expression studies using either labelling with [^35^S] methionine (left part) or [^35^S] cysteine (right part) and immunoprecipitation. The names of the constructs are given on top of the gel. Vacc: negative control showing products precipitated from mock transfected vaccinia virus MVA-T7 infected cells.

Despite the variability of the results obtained by the deletion or introduction of stop codons or the effects resulting from different positioning of the stop codons in the region downstream of the start/stop site, it was tempting to speculate that the availability of downstream stop codons controls the frequency of reinitiation. A reasonable working hypothesis was that ribosomes reading through the stop codon #1 in the start/stop site, maybe enhanced by the 18S rRNA interaction with the TURBS, terminated at downstream inframe termination signals and could thereby be made available for reinitiation. If this theory was correct, a significant amount of the ribosomes synthesizing VP1 should pass beyond stop codon #1 and terminate at stop codon #2 or #3. To test for this hypothesis, we established a set of constructs, in which the cysteine codons in the VP1 coding frame were mutated and new cysteine codons were created in the region downstream of the termination signal of the start/stop site (stop codon #1) (Fig. 6). Detection of VP1 expressed from these constructs in the presence of ^35^S Cys would demonstrate translational readthrough at stop #1. When the latter termination signal was present, VP1 could no longer be labelled with ^35^S Cys (constructs pMH168 and pMH170) whereas Cys labelling could be demonstrated for the control with a mutation inactivating the original termination signal of the VP1 coding sequence (pMH169). These results showed that the effects on VP2 expression levels observed after addition, removal or movement of termination codons in the region downstream of the start/stop site were not due to a translation termination function executed by these sequences.

### Search for secondary structure motifs modulating VP2 expression

**Figure 7:**
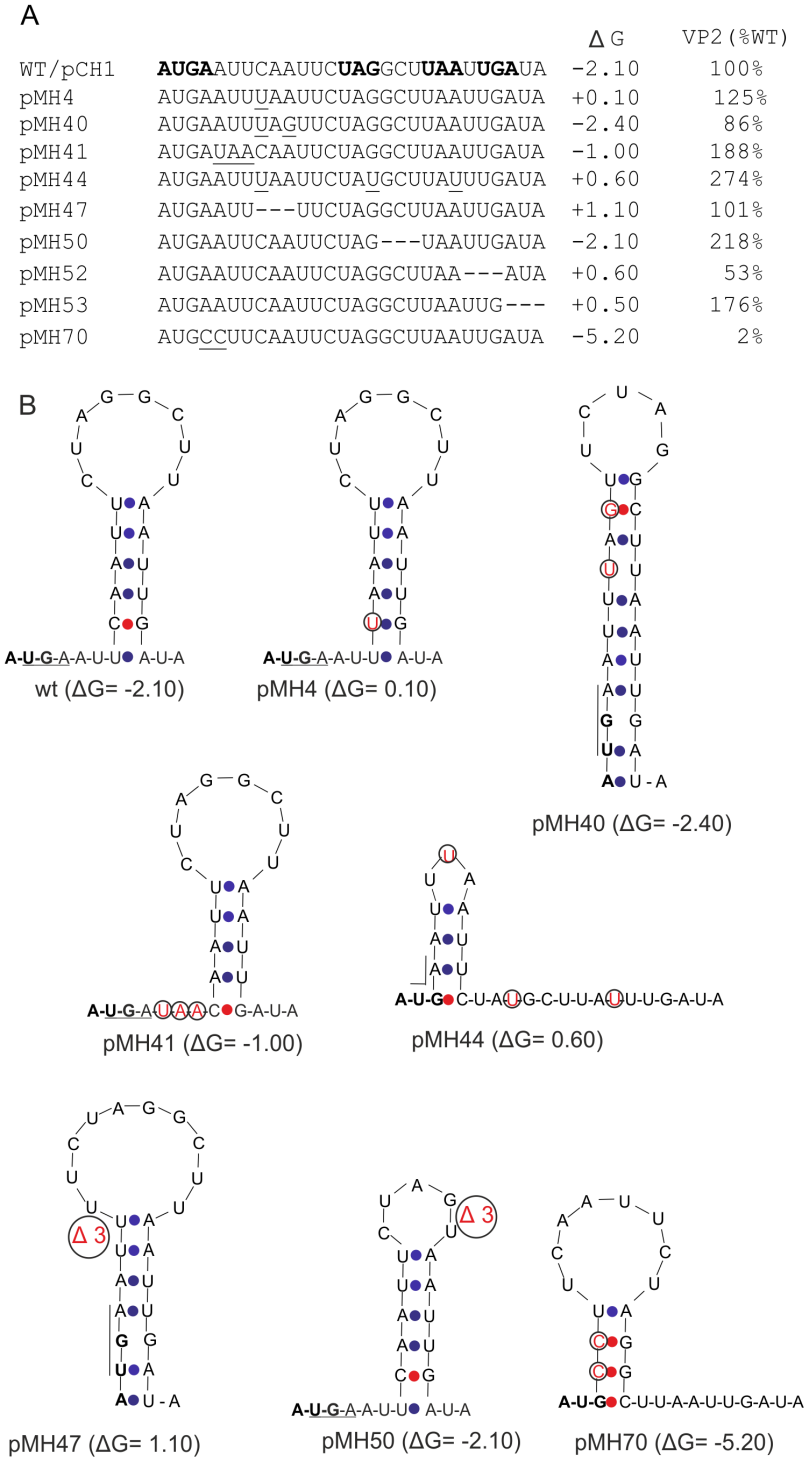
Ability for hairpin structure formation in the start/stop region of wt and mutant sequences. A) Sequences of the 5′ terminal region of the VP2 coding RNA derived from the wt construct pCH1 or mutants thereof displaying the indicated changes. Mutated nucleotides are underlined and deleted residues are indicated as horizontal lines. In the wt sequence the start/stop residues as well as termination codons downstream thereof are given in bold face. On the left site, the name of the cDNA constructs is given whereas on the right the free energy of the secondary structure (determined by Mfold, given as ΔG in kCal/mol) and the VP2 expression efficiency as percent of the wt level is given. B) Mfold calculated structures of a selected set of the RNAs given in (A). The start codon of the start/stop site is given in bold face, the stop codon is highlighted by a line. Nucleotides differing from the wt sequence are given in red and circled. A-U or G-U pairing is indicated by blue dots, G-C pairing by red dots.

**Figure 8:**
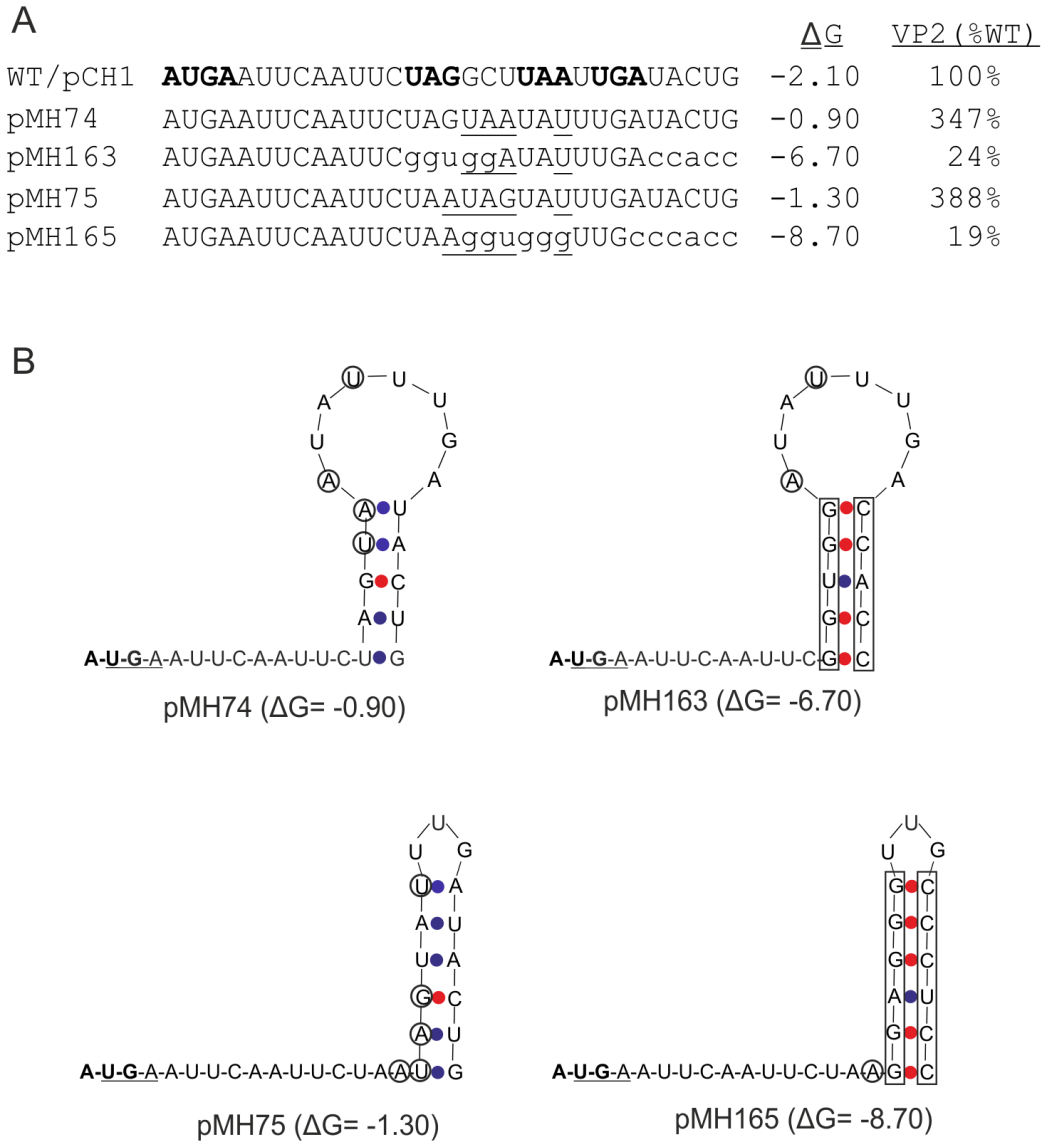
Test for a correlation of hairpin stability with reinitiation frequency. A) Sequences of the 5′ terminal region of the VP2 coding RNA derived from the wt sg mRNA encoding construct pCH1 or mutants thereof displaying changes affecting stability of putative hairpin structures closely downstream of the start/stop region. Mutated nucleotides are underlined. Differences in mutants pMH163 and pMH165 with regard to mutants pMH74 and pMH75, respectively, are given in lower case. For the wt construct pCH1 the start/stop region and termination codons downstream thereof are given in bold face. Left of the sequences, the names of the constructs are given, whereas on the right side the stability of the secondary structure (determined by MFold and given as ΔG in kCal/mol) and the VP2 expression rate (given as mean value of at least three independent experiments in % of the wt level which was set to 100%) are shown. B) Mfold calculated structures of the RNAs derived from the constructs given in (A). The start codon of the start/stop site is given in bold face, the stop codon is highlighted by a line. Nucleotides differing from the wt sequence are circled or marked by a box when a stretch of several nucleotides is affected. A-U or G-U pairing is indicated by blue dots, G-C pairing by red dots.

The mutation analysis of the 5′ region of the VP2-coding sequence revealed a significant modulation of VP2 expression levels by this region. However, there was no clear clue to the observed effects coming from primary sequence analysis. It was therefore obvious to look for secondary structure motifs that might be pushing or hindering reinitiation. Using Mfold for modelling the RNA secondary structure of the sequence, a hairpin structure located two nucleotides downstream of the start/stop side was identified (Fig. 7). In a second step, we analyzed a selected set of our mutants with Mfold for hairpin structures, their location and stability. As shown in Fig. 7 all analysed sequences were able to form hairpin structures in close vicinity to or even overlapping the start/stop site. However, neither location nor stability of the hairpins correlated in a decent way with the previously determined expression levels. The only striking result was the significantly higher stability of the hairpin in pMH70 that was accompanied by a very low VP2 expression level. This could mean that beyond a threshold level of a downstream hairpin stability, estimated somewhere between ∼2.5 and −5.2 kcal/Mol, reinitiation might be blocked. To test for this hypothesis, we established a pair of mutants based on the constructs pMH74 and pMH75 that had shown considerably increased VP2 levels (Fig. 5). Mfold analysis indicated for these constructs rather unstable hairpins located further downstream compared to the one found in the wt construct (Fig. 8). To obtain more stable hairpins at the same sites as in the parental constructs, appropriate changes were introduced into the stem regions giving rise to pMH163 and pMH165, respectively. The resulting hairpin structures exhibited both significantly increased stability of −6.7 and −8.7 kcal/Mol compared to the structures in the parental plasmids (−0.9 and −1.3 kcal/Mol, respectively). For both constructs, considerably reduced VP2 expression of 24% and 19% of the wt level was determined. These findings support the hypothesis of hindered reinitiation resulting from secondary structures with increased stability closely downstream of the start/stop site.

### Effects of mutations modulating VP2 expression on virus replication

The biological significance for using reinitiation of translation for expression of the minor capsid protein VP2 in caliciviruses is not known. One possible explanation is that provision of a defined ratio of VP1 and VP2 is necessary or at least preferable for efficient virus propagation. The data presented above showed that the primary and secondary structure of the region downstream of the start/stop site modulates the reinitiation frequency in FCV. A selected set of modulating mutations was used to address the question whether a defined VP2 expression rate is important for FCV propagation. To this end, the mutated sequences were introduced into the infectious FCV cDNA construct. The resulting plasmid DNA was subsequently used for transfection of CRFK cells infected with vaccinia virus MVAT7 for virus recovery. After removal of the vaccinia virus, supernatant from the transfected cultures was used for infection of fresh feline cells (CRFK cells) [27].

**Figure 9:**
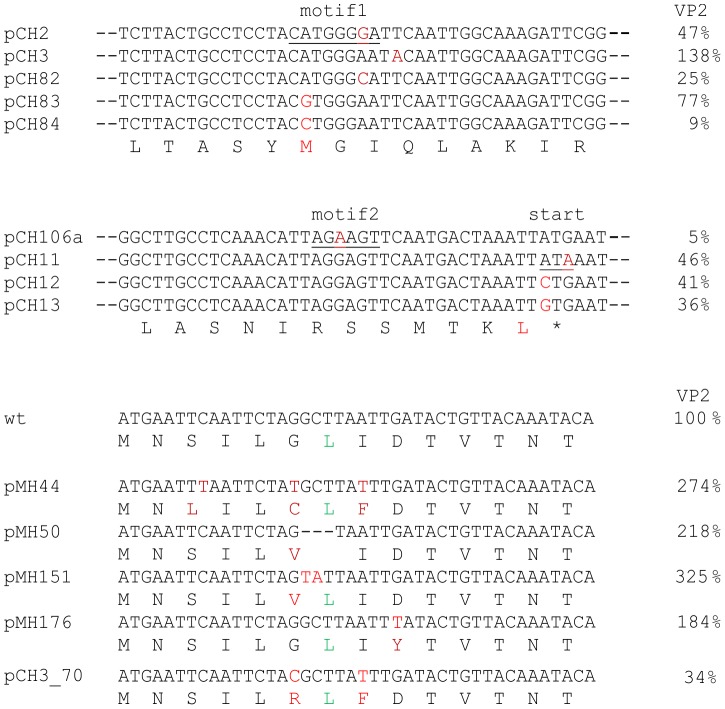
Recovery and analysis of viruses with mutations altering VP2 expression rates. In the upper part, the cDNA sequence of part of the TURBS region is shown for the constructs specified on the left with the encoded amino acid sequence of wt given in the bottom line. Since the introduced mutations are concentrated in different regions only these parts of the sequence harbouring exchanges are shown resulting in two different blocks. Nucleotide exchanges are highlighted in red. In the amino acid sequence, the two residues that are changed in mutants pCH83/pCH84 or pCH12/pCH13 are shown in red. In the lower part, the sequences coding for the aminoterminal part of VP2 of a third group of constructs with exchanges in this area are shown. Below each nucleotide sequence, the encoded amino acid sequence is given. Changes with regard to the wt sequence are given in red. The Leu (L) at position 7 of VP2 is shown in green since this position was shown before to be critical for virus viability [16]. On the left, the names of the constructs are given whereas on right site the VP2 expression efficiencies in per cent of the wt level are indicated (given as mean value of at least three independent experiments).

We selected mutants from the above described set as well as mutants described previously [23]. In addition to changes affecting the region downstream of the start/stop site, we also included mutations of TURBS motif 1, TURBS motif 2 and the start codon (Fig. 9). The VP2 expression levels of the selected mutants determined before in the transient expression studies ranged from 5 to 325% of the wt level. Virus was recovered for 7 out of 13 tested mutants (Tab. 1). Sequencing RT-PCR products amplified with RNA from passage #6 of the viruses revealed that one of the recovered start codon variants (derived from construct harbouring the pCH13 mutation) had reverted and the second one (pCH11 mutation) displayed a mixture of revertant and mutant indicating instability of these changes in the context of the replicating virus. The respective viruses were not further analysed.

**Table 1:** Results of virus mutant recovery experiments.

	VP2% (transient)	Virus CPE	Seq P1	Seq P6	Growth	VP2% (Virus)	Trans-Compl.: virus
pCH2	47	+	+	+	∼wt	36	
pCH3	138	+	+	+	∼wt	99	
pCH11	46	+	+	+/wt			
pCH13	36	+	+	wt			
pCH82	25	−					+^1^
pCH83	77	+	+	+	def	81	
pCH84	9	−					+^1^
pCH106a	5	−					+^1^
pMH44	274	−					−2
pMH50	218	−					−2
pCH3_70	34	−					+^2^
pMH151	325	(+)	V->E	V->E		97 (E)	− (V)^2^
pMH176	184	+	Y->H	Y->H		92 (H)	− (Y)^2^

A selected set of mutations was introduced into the FCV infectious cDNA clone and tested for virus recovery as described before [27]. The left column lists the names of the constructs (based on pCH1), into which the respective mutation was first introduced and analyzed in transient expression assays. In the second column, the VP2 expression level determined in the transient analyses is given as percent of the wt level. The third column gives the results of the virus recovery experiments with ‘+’ representing detection of CPE after transfer of supernatant from the transfected to fresh CRFK cells, ‘(+)’ representing a questionable outcome and ‘−’ indicating clearly negative results. Seq P1 and Seq P6 (columns 4 and 5) indicate whether the introduced mutation was still present in the viral RNA after 1 or 6 passages, respectively. Presence of the mutation: ‘+’; reversion to wt sequence: ‘wt’; mixture of mutation and wt: ‘+/wt’; change of the mutated residue to another non-wt amino acid is given in the one letter code. In column 6, the growth characteristics of the recovered viruses are given with ‘∼wt’ representing wild type like growth and ‘def’ indicating significant growth retardation and reduced maximal titer. The second last column presents the VP2 expression levels determined for cells infected with the recovered viruses (3 to 5 independent experiments). The right column shows the results obtained in a *trans* complementation assay (as described in [16]) with ‘+’ showing successful *trans* complementation with the mutant VP2, and ‘−’ indicating a failure to complement. For the constructs with secondary mutations (two bottom lines) the secondary mutants were present in the recovered viruses, whereas the original VP2 mutant was used for complementation as indicated in the right two columns. Empty fields indicate that the respective analysis has not been done.

1 Transcomplementation assay was conducted with co-transfection of mutated full length clone and wt VP2 expression construct.

2 Transcomplementation assay was conducted with co-transfection of full length clone containing 3 stops in the VP2 coding sequence and VP2 expression construct containing the analyzed mutations.

Two more mutants, that both yielded higher VP2 levels than the wt, showed no reversion but a change of the altered sequence (pMH151 mutation: original change V for G, passage 6 virus E for V and pMH176 mutation: original change Y for D and passage 6 virus H for Y). To analyse, whether the original mutations interfered with virus propagation, we tested these parental mutants in a transient *trans* complementation assay. To this end, VP2 expression from the full length construct was blocked by the introduction of three stop codons in the VP2 coding sequence as described previously [16] and VP2 displaying the desired mutations was expressed from a second plasmid via the T7 promoter and vaccinia virus MVAT7. In this setup, the sequence, from which VP2 is expressed, is not replicated and transcribed by the viral RNA polymerase so that reversion to wt sequence by mutation is excluded. Nevertheless, significant amounts of (mutated) VP2 are present and allow recovery of infectious virus that can be detected upon infection of fresh cells with supernatants of the transfected cultures via observation of CPE. Complementation of the FCV genome containing the three stop codons by *in trans* expression of wt VP2 was easily achieved showing the suitability of the test system. The same experiment for *in trans* complementation conducted with VP2 bearing the MH151 or MH176 mutations gave a clearly negative result. Since the only difference between these assays and the positive control was the presence of a single mutation in the VP2 protein provided *in trans* the presence or absence of CPE could only be due to successful or failed complementation and thus showed that the originally introduced mutations in MH151 and MH176 were indeed deleterious. Thus, virus recovery described above for the MH151 and MH176 mutants was obviously dependent on the secondary change of the altered sequence. These secondary changes reduced VP2 expression to about wt levels (Tab. 1).

Equivalent *trans* complementation assays were conducted for the mutants harbouring the MH44 and MH50 mutations in VP2 (Fig. 9). The respective mutations had been found to lead to increased VP2 levels but did not yield infectious viruses without complementation (Tab. 1). As expected, *trans* complementation of these constructs with the mutated VP2 proteins did not lead to virus recovery supporting the conclusion that the introduced changes of the VP2 sequence were deleterious.

Virus mutants with stable alterations were recovered from full length constructs pgCH2 and pgCH83 displaying motif 1 changes that had been found before to reduce VP2 expression levels to 47% and 77% of the wt-level, respectively (Fig. 9 and [23]). These results show that a reduction of the VP2 amount within the infected cell by about 50% is not critical for virus recovery and propagation.

Three further mutations affecting motif 1 and motif 2, that were originally analyzed in constructs pCH82, pCH84 and pCH106a [23], were found to provide a severe reduction of VP2 expression levels to 25, 9 and 5%, respectively. The pCH84 mutation leads to a conservative amino acid exchange in VP1 (M648L) whereas the changes in the other two constructs are silent. Accordingly, the effects of these mutations on virus viability were the most important to be tested, since viral protein function should not (pCH82 and pCH106a) or not severely (pCH84) be affected by these changes so that the effect of the changed VP2 concentration should be dominant. When tested in the full length construct, all three changes prevented virus recovery (Tab. 1). In some experiments, CPE was detected very late after transfer of the transfection lysate to fresh CRFK cells. The sequences of these recovered viruses showed reversion indicating that FCV containing these mutations was not able to produce progeny virus. To obtain further support for the conclusion that the low level of VP2 protein obtained from the mutated sequences was responsible for this result, these constructs were tested once again in the *trans* complementation assay (Tab. 1) [16]. In contrast to the approach described for pMH151 and pMH176 above, the complementation test was in these cases conducted by simply providing wt VP2 *in trans* by co-transfection of the full length clone carrying the original mutation in the VP1 coding region and a VP2 expression construct displaying the wt sequence. This setup increases the amount of VP2 in the transfected cells but preserves the VP1 mutation. All three mutants produced significant amounts of infectious viruses upon transfection as could be concluded from detection of CPE early after transfer of the transfection lysate to the CRFK cells. Thus, in these three constructs indeed the reduced amount of VP2 expressed from the mutants prevented recovery of infectious virus.

Similarly, a full length construct based on mutant pCH3_70 with mutations in motif 1 and the stop codons #2 and #3 in the VP1 frame downstream of the start/stop site (Fig. 9) didn't produce viable viruses. For this mutant, VP2 expression had been shown before to be reduced to ca. 34% of the wt level. The mutations result in a VP2 sequence altered at two positions (G6R; I8F). Accordingly, the defect in virus production could either result from a non-functional VP2 or from insufficient amounts of the protein. To test for these two alternatives, we conducted a *trans*-complementation assay, in which the full length construct containing the 3 stop codons in the VP2 frame was transfected together with a plasmid expressing VP2 with the G6R/I8F mutations. In this setup, in which the amount of the mutated VP2 is raised due to the vaccinia virus based expression, virus recovery was observed. Thus, again the amount of VP2 obtained from the mutant full length construct and not the two exchanges in VP2 were responsible for the defect in virus recovery observed before in the experiment without *trans* complementation. Taken together, the experiments revealed that VP2 levels below ∼34% of the wt level prevent production of infectious FCV.

**Figure 10:**
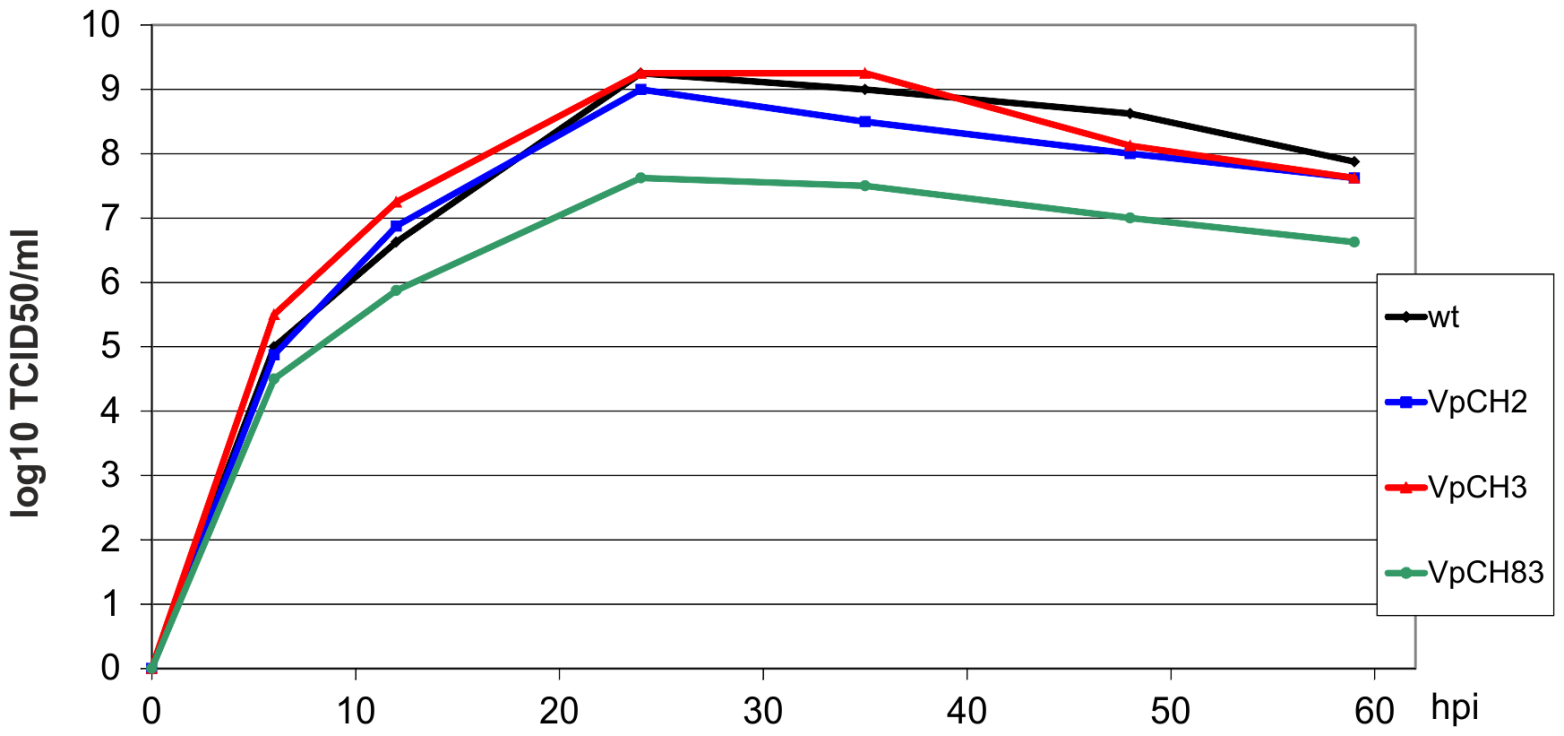
Growth curves of viruses with TURBS mutations. Growth curves of viruses recovered from the full length constructs containing the mutations displayed by pCH2, pCH3 and pCH83 (VpCH2, VpCH3 and VpCH83, respectively) in comparison to the virus recovered from the wt infectious cDNA clone. The curves show the results of a representative experiment in titer (TCID_50_) at a given time point (hours post infection – hpi). The infection was done at an m.o.i. of 0.0003.

For quantitative analysis of the influence of altered VP2 levels on virus propagation, we recorded growth curves of the recovered viruses (Fig. 10). VpCH2 and VpCH3, the two viruses harbouring the mutations originally tested in pCH2 and pCH3, respectively, replicated with kinetics and maximum titres very similar to the wt. In contrast, VpCH83 displaying a mutation in motif 1 leading to a M to V amino acid exchange and a moderate reduction of VP2 expression to 77% of wt level [23] was clearly hampered with a maximum titre reduced by more than 1.5 log10 units compared to the other three tested viruses. This result is most probably not due to the moderately reduced VP2 expression but to the amino acid exchange since VpCH2 replicated as well as wt with significantly lower amounts of VP2. Taken together, these data show that reduction of the amount of VP2 below ca. 30% of wt level interferes with production of infectious FCV whereas ca. 50% of the normal amount is sufficient for wt-like replication.

### Expression of VP2 from a separate mRNA results in viable virus

**Figure 11:**
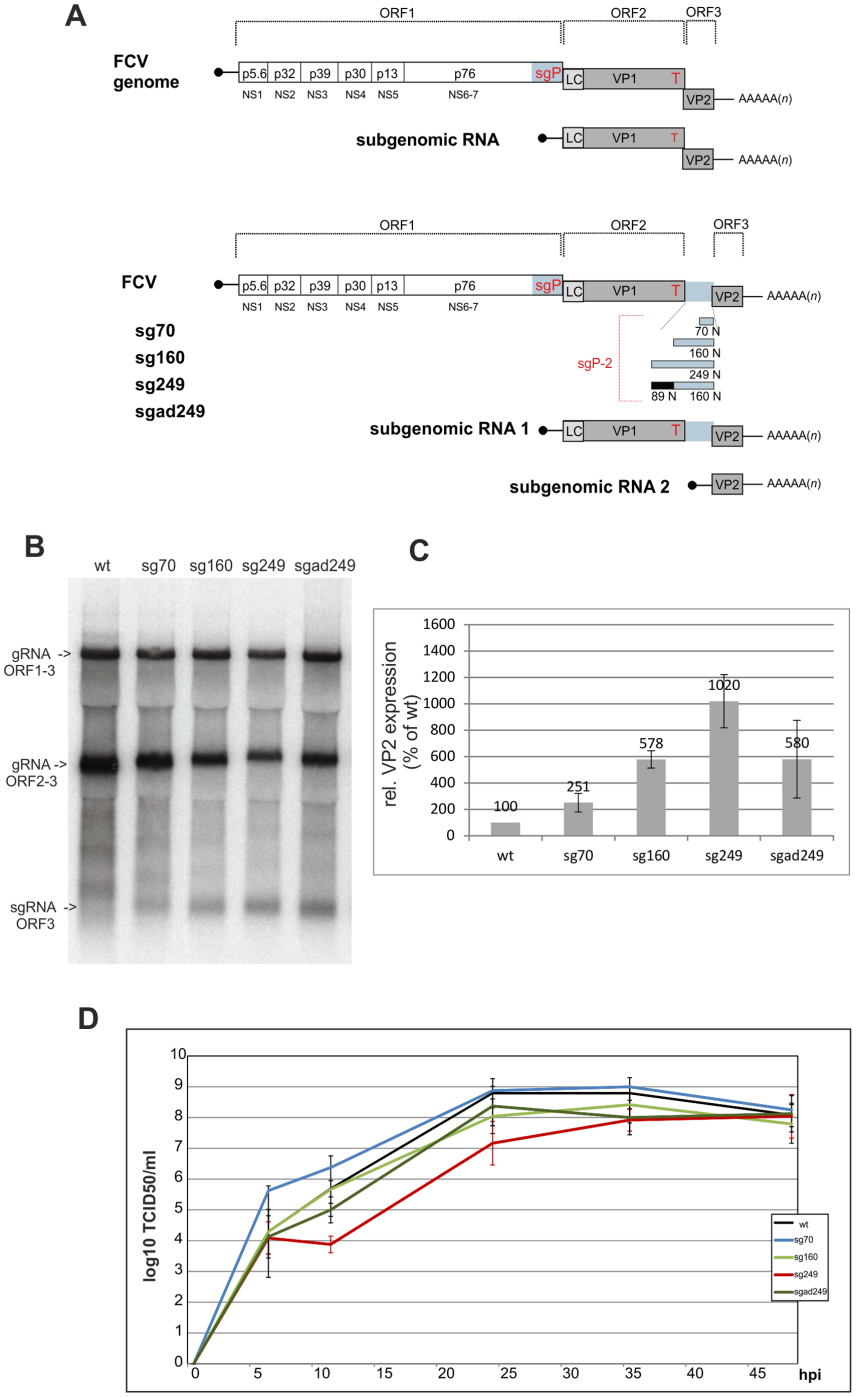
Construction, and analysis of viruses expressing VP2 via a separate sg mRNA. Establishment and characterization of viruses containing in their genomes duplicated sg mRNA promoters. (A) Genome organization of the different viruses is given as a scheme (not drawn to scale). Grey bars represent ORF2 and ORF3 coding for the capsid proteins and the leader of the capsid protein (LC, light grey). The polyprotein containing the nonstructural proteins (encoded by ORF1) is symbolized by a white bar with the designation of the different cleavage products. Black lines represent the nontranslated regions (NTR) at the 5′ and 3′ end, the 3′ poly(A) tail is indicated. VPg (virus protein, genome linked) present at the 5′ ends of both the genomic and subgenomic RNAs is symbolized by a black circle at the end of a line representing the 5′ NTR of the RNAs. The proteins encoded by the subgenomic RNA are indicated by abbreviations: LC: Leader of the capsid, VP1: major capsid protein, VP2: minor capsid protein. The red ‘T’ stands for the TURBS. The sg promotor region and duplicated fragments thereof are shown in light blue, a nonrelated sequence used as a spacer in sgad249 is represented by a black bar. (B) Northern blot with RNA isolated from cells infected with the viruses indicated above of the gel. Hybridization with a probe covering the VP2-coding sequence was done as described before [27] (C) The VP2 expression rate of the recovered viruses was determined by *in situ* labelling with ^35^S amino acids, immunoprecipitation and phosphorimager analysis. The results are shown as percent of the wt virus expression rate (set to 100%). The values were normalized to the VP1 expression rate. Error bars represent standard deviation determined from at least 3 independent experiments (D) Growth curves of the recovered viruses inoculated at a starting m.o.i. of 0.0003. The graph shows the mean values of 2 to 3 experiments with the standard deviation indicated by error bars.

One possible explanation for the use of translation reinitiation in caliciviruses could be the establishment of a certain VP1∶VP2 ratio required for some unknown reason for productive virus replication. The analyses described above allowed definition of a VP2 threshold level essential for recovery of infectious progeny virus. Unfortunately, mutations leading to considerably higher VP2 expression levels reverted quickly due to non-functional VP2 as demonstrated by the *trans* complementation assays. To obtain virus mutants with significantly enhanced VP2 expression, we decided to construct full length plasmids, in which VP2 should be expressed from a duplicated sg mRNA promoter. It was shown before that the sg mRNA transcription in RHDV is driven by a region located upstream of the genomic position corresponding to the 5′ end of the sg mRNA [30]. We therefore inserted fragments of 70, 160 and 249 nucleotides derived from the respective region into the FCV genome at the reinitiation start/stop site, thereby separating the VP1 and VP2 genes by a duplicated putative sg promoter placed upstream of the VP2-coding sequence (Fig. 11A). Attempts to recover virus from all three of these constructs were successful. Northern blots proved the transcription of a second subgenomic mRNA for the viruses containing a duplicated promoter with the intensity of the band increasing with the length of the duplicated fragment (Fig. 11B). Protein analyses based on *in situ* labelling of polypeptides with ^35^S amino acids, immunoprecipitation, gel electrophoresis and phosphoimager analysis revealed the presence of VP2 levels of 251, 578 and 1020% of the wt for the duplicated 70, 160 and 249 fragments, respectively, showing that FCV can tolerate a tenfold overproduction of VP2 (Fig. 11C).

In a next step we tested the growth properties of the recovered viruses in more detail. Growth curves were recorded showing that the viruses containing the 70 or 160 nucleotide duplications exhibited growth properties very similar to the wt virus, whereas the virus with the 249 nucleotide insertion replicated significantly more slowly (Fig. 11D). The latter result could either be due to the length of the insertion or it could result from VP2 levels exceeding a tolerated maximum. To be able to discriminate between these two possibilities, we established construct sgad249, which contained the 160 N duplication of the sg promoter region preceded by an additional unrelated 89 N fragment resulting in a total extra sequence of 249 nucleotides. Again a recombinant virus was easily recovered and expressed VP2 to levels comparable with the 2_sg160 derived virus (580 versus 578% of wt level). Thus, the degree of VP2 expression enhancement is indeed dependent on the length of the duplicated promoter fragment and not on the total insertion size. More importantly, the growth curve of the sgad249 derived virus was more similar to the 2_sg160 than to the 2_sg249 virus.

## Discussion

Reinitiation of translation upon terminating the synthesis of a long protein is a rare event in eukaryotes [22], [23], [31]–[38]. Expression of the minor capsid protein VP2 of caliciviruses follows this scheme, and was the first case, for which the mechanism of such a reinitiation event was elucidated in more detail. In summary, a region upstream of the start/stop site where the ORFs coding for the major capsid protein VP1 and for VP2 overlap is of major importance for this process. This special sequence element is called the ‘termination upstream ribosomal binding site’ (TURBS) since it was shown to contain a sequence hybridizing with part of the 18S rRNA of the small ribosomal subunit. This interaction is believed to tether the terminating ribosome to the RNA, thereby giving time for reloading of initiation factors necessary for reinitiation of translation [18]. In addition, motif 1 was shown to interact with eIF3 [22]. The sequences of two further motifs, motifs 2* and 2, are complementary to each other and thought to form a stem structure necessary for presentation of motif 1. The arrangement of the TURBS motifs leads to positioning of the ribosome at the start site making reinitiation independent of a canonical AUG start codon. Some of the features of the TURBS element, especially eIF3 binding, binding and positioning of the small ribosomal subunit at the start site are reminiscent of features characteristic for the HCV or pestivirus IRES [39]–[43]. However, there is no evidence that a TURBS can function as an IRES.

Although significant effort has been made to investigate the molecular basis of translation reinitiation in some viral RNAs [18]–[23], [31], [33], [35], [36], [44]–[47], many aspects of these processes are still obscure. Data from earlier studies on the caliciviruses RHDV and FCV indicated that sequences downstream of the known motifs play a role in reinitiation [19], [20], [23]. One of these findings was the significant reduction of reinitiation frequency in RHDV in consequence of changing the termination codon in the start stop site from UGA to UAA or UAG [19]. This result cannot be explained by the present knowledge of the TURBS and the reinitiation process. This is also true for the fact that mutation of the two termination codons in the VP1 coding ORF located downstream of the start/stop site in FCV at codon positions 4 and 6 with regard to the first stop results in a reduction of VP2 expression level to 42% [23].

The above described observations point towards a role of sequences downstream of the start/stop site for reinitiation. To analyse this in detail, we first established a set of mutants containing 3mer deletions in the first 36 residues downstream of the start/stop site in construct pCH1 [23]. Determination of the VP2 expression levels normalized to the VP1 yield revealed that some mutants exhibited altered VP2 levels in a range of ca. 50 to more than 200% of wt level. In subsequent analyses with a variety of point mutations affecting the first nucleotides downstream of the start/stop site we showed that some of these mutations severely hampered or even almost abrogated VP2 expression. The most prominent effects were seen for the constructs pMH30, pMH70, pMH145 and pMH7, in which at least the third base of the TGA stop codon at the end of the VP1 coding sequence and the following A residue were changed. These alterations reduced VP2 expression to 2–36% of wt level. This effect is not due to loss of the translational stop signal since other mutations destroying this function reduced VP2 expression only to levels ranging from 71 to 91% (pMH142, 143 and 144 in Fig. 4 and pCH14, 15 and 16 in [23]). Similarly, the residue following the termination codon alone is not of major importance, since single nucleotide exchanges at this position resulted in reinitiation rates of 51 to 136% (pMH1, 2 and 69 in Fig. 4). Most importantly, exchange of these two nucleotides had not in all cases a severe effect on VP2 expression. Construct pMH31 with a transversion of the two respective A residues to T showed only a mild reduction of the VP2 amount to 73%. Taken together, these two residues have an influence on VP2 expression efficiency, but the effect of mutations is not *per se* dependent on loss of the two A nucleotides and varies with the sequence replacing the AA.

We had already seen before that mutation of the two downstream termination codons following the UGA in the start/stop site resulted in reduced VP2 expression (42%, [23]). To analyse whether this effect is due to loss of a putative role as functional termination signals, we established and tested a large variety of constructs with mutated or moved stop codons, or introduced additional termination signals with different sequences at various sites. The VP2 expression levels determined for these constructs ranged from 24 to more than 520% of wt level but we were not able to correlate decrease or increase of VP2 expression with number, position or sequence of the termination signals. Moreover, an experiment designed to test whether these additional stop codons might be functional as additional termination signals for stopping read through of ribosomes of the first termination signal in the VP1 frame did not yield any data supporting this idea. Thus, the results obtained here again prove the importance of the first part of the VP2 coding sequence for reinitiation efficiency but do not shed light on the underlying mechanism.

The results discussed above exclude a simple primary sequence based function of the region downstream of the start/stop site for reinitiation. It therefore seemed logical to look for secondary structure elements containing (part of) the respective sequence. A computer-based search revealed potential for hairpin structure formation in the respective region not only in FCV but also in RHDV (not shown). Different mutations affecting stability and localization of the hairpin significantly affected VP2 expression. Unfortunately, the results obtained in these experiments could again not be correlated with the above mentioned features of the hairpin. Secondary structure analyses of the TURBS region have been conducted before for the FCV, the Influenza B virus and the MNV TURBS via *in silico* and in the latter cases also biochemical analyses [21], [22], [46], [47]. An obvious conserved secondary structure fold was not identified and only little information is available for the structure/function relationship of the TURBS secondary structure. Similar to the FCV RNA, the MNV RNA exhibits a stable hairpin (ΔG = −5.2 kCal/mol) a few nucleotides downstream of the start/stop site, whereas sequences downstream of the start/stop site in the Influenza B virus TURBS seem to hybridize with a region upstream of motif 2*. Importantly, the function of the sequences downstream of the start/stop site has not been investigated for these viruses. For the double-stranded RNA fungal virus Helminthosporium victoriae virus 190S (genus *Victorivirus*, family *Totiviridaecase*) a different type of coupled translation reinitiation not relying on a TURBS has been described [48]. In this case, RNA structure requirements for translational restart have been determined on a functional level and were defined to represent a pseudoknot upstream of the restart site. A similar structure was not found in caliciviruses.

Taken together, the set of data presented here shows that the 5′ region of the VP2-coding sequence definitely influences reinitiation. Mutations in this region can abrogate VP2 expression (2% residual activity in pMH70) or strongly enhance reinitiation (more than 500% in pMH153). However, the observed modulation of VP2 expression depends on the individual residues at specific positions, so that we were not able to deduce from our results a clear pattern in primary sequence or secondary structure that promotes or hampers VP2 expression.

So far, the biological meaning of translation reinitiation for calicivirus VP2 expression is obscure. It has been shown that the minor capsid protein VP2 is essential for production of infectious virus particles [16]. These authors have also shown that VP2 can be provided *in trans*, so that coupling of VP1 and VP2 synthesis cannot be the reason for employing reinitiation for VP2 expression. To check, whether the amount of VP2 present in an infected cell plays an important role for virus recovery, we established a set of mutants in our infectious cDNA clone [27]) with alterations affecting the VP2 expression level. In a first experimental approach we were able to recover several stable virus mutants that displayed VP2 expression levels of ca. 50 to 140% of wt. Growth curves showed for these viruses replication rates very similar to the wt virus. Accordingly, these rather mild changes of VP2 levels had no significant impact on virus growth.

Mutants pCH82, pCH84 and pCH106a contain nucleotide exchanges that were shown before to reduce VP2 expression to 5, 9 and 25% of wt (pCH106a, pCH84 and pCH82, respectively). Viable viruses could not be recovered from the full length constructs harbouring these changes although the introduced mutations were silent (82 and 106a) or conservative (84, M648L), so that impaired function of mutant proteins could be excluded or was very unlikely (82 and 106a or 84, respectively). Accordingly, it seemed rather evident that the VP2 amount expressed from these mutants was too low to support virus recovery. This conclusion was strongly supported by the fact that virus particles could be recovered from these full length constructs and passaged to fresh cells when VP2 was provided *in trans*. Similarly, virus could be recovered from the construct containing the CH3_70 mutations (two amino acid exchanges in VP2 and a motif 1 mutation), when the mutated VP2 was provided in higher amounts *in trans*. Since the CH3_70 mutations lower the VP2 expression to 34% it can be concluded that a VP2 expression rate of ca 30% or less of the wt level is deleterious for virus recovery in our system.

Caliciviruses use a mixed strategy for expression of their proteins, namely translation and proteolytic processing of a polyprotein (non-structural proteins and in vesiviruses also L-VP1 precursor), transcription and subsequent translation of a subgenomic mRNA as well as leaky scanning (only MNV) and reinitiation of translation. Since all necessary prerequisites for expression of subgenomic mRNAs are available, VP2 expression could in analogy with the situation in members of the *Nidovirales* also be managed via a further mRNA co-linear with the last ca. 300 nucleotides of the genome. For unknown reasons, evolution of caliciviruses has not gone into this direction, but we show here for the first time that such an arrangement is in principle functional. Our data show that the insertion of a fragment representing a duplication of the 70 3′ terminal nucleotides of ORF1 between ORF2 and ORF3 is sufficient for transcription of a smaller second 3′ terminal mRNA that is translated to give rise to VP2. Compared to the wt virus, the resulting recombinant expresses about 2.5 times more VP2 and grows perfectly well. Even higher amounts of VP2 were obtained when larger fragments derived from the original sg promoter were inserted between ORF2 and ORF3. A fragment of 160 nucleotides yields ca. 5.8 times more VP2 than wt, whereas a 249 residue promoter fragment produces roughly 10 times more VP2. The increase of sg mRNA synthesis with longer promoter sequences differ from what was found in an *in vitro* assay for RHDV where a 50 nucleotide fragment was sufficient to provide full sg promoter activity [30]. This discrepancy might result from the different assay systems or might reflect differences between lago- and vesiviruses. A systematic *in silico* search for secondary structures in calicivirus genomes revealed the presence of a stable hairpin in the genomic negative strand located within the region corresponding to the sequence duplicated in our constructs [49]. In the case of vesiviruses, the putative hairpin is made up by roughly 60 nucleotides, located six nucleotides downstream of the sg mRNA transcriptional start site in the negative strand copy of the genome. Since use of a fragment containing the complete hairpin is by far not sufficient to ensure transcription initiation at the highest possible efficiency, sequences further downstream in the negative strand RNA have to play a role for sg mRNA transcription. Nevertheless, conservation of presence and location of the hairpin among caliciviruses resulted in the plausible proposition that it plays a role in sg promoter function [49]. This tempting hypothesis has to be investigated in detail experimentally. This and further work is needed to identify the essential motifs in the FCV sg promoter and elucidate the molecular basis of polymerase binding.

The virus with the 249 nucleotide sg promoter insert was found somewhat hampered leading to mildly reduced growth rates. This reduction is either due to the overproduction of VP2 or to specific features of the duplicated sequence but not to the size of the insert, since an insertion composed of the 160 nucleotide sg promoter fragment and a nonrelated sequence of 89 residues behaved like the sg160 mutant both with regard to VP2 expression level and growth rate. Even though an effect of the 5′ part of the duplicated sequence in the virus with the sg249 insertion cannot be excluded, it is likely that its reduced growth rate is due to excess of VP2. It therefore seems likely that there is also an upper level of VP2 concentration within the infected cell that can be tolerated. VP2 amounts exceeding this upper level impair virus replication. However, a VP2 level within defined but rather broad limits could also be obtained by other expression strategies.

An interesting question for future work is what drives VP2 inclusion into virus particles and whether increased VP2 expression levels result in increased incorporation of the protein into virions which might even reduce stability of the capsid. So far, only rather vague indications have been found for putative functions of the protein. Negative regulation of the activity of the RNA polymerase in Noroviruses [12], influence on expression and stability of norovirus VP1 [13], possible interaction with VP1 and in connection with this feature assistance in genome packaging [14] have been described. For MNV a role of VP2 in regulation of the maturation of antigen presenting cells has been shown that could have major impact on the adaptive immune response to virus infection [15] This role of VP2 for interaction of the virus with its host might also be relevant for other caliciviruses. However, there is so far no clear evidence for the need of VP2 for productive calicivirus replication in tissue culture cells. More insight into the essential function of VP2 in the context of virus replication is urgently needed to better understand the importance of the special expression system used for this protein.

## Conclusions

We show here that the sequence context downstream of the start/stop site in FCV RNA modulates reinitiation frequency in a range of more than two orders of magnitude. An elaborate set of data did not allow to deduce a clear systematic behind these findings so that the observed effect on VP2 translation has to be regarded as position and nucleotide specific. Introduction of various changes affecting the VP2 expression rate into the viral genome revealed that FCV can tolerate rather gross changes of VP2 concentrations but with obvious minimal and maximal limits beyond of which virus propagation is considerably hampered.
