# Correction: Feline Calicivirus Can Tolerate Gross Changes of Its Minor Capsid Protein Expression Levels Induced by Changing Translation Reinitiation Frequency or Use of a Separate VP2-Coding mRNA

**DOI:** 10.1371/journal.pone.0107081

**Published:** 2014-08-25

**Authors:** The PLOS ONE Staff

## Notice of Republication

This article was republished on August 7, 2014, to replace incorrectly changed characters in the byline, citation, copyright statement, and Acknowledgments. The publisher apologizes for the errors. Please download this article again to view the correct version. The originally published, uncorrected article and the republished, corrected article are provided here for reference.

## Supporting Information

File S1 Originally published, uncorrected article.(PDF)

File S2 Republished, corrected article.(PDF)
